# Photosynthetic energy conversion efficiency in the West Antarctic Peninsula

**DOI:** 10.1002/lno.11562

**Published:** 2020-07-20

**Authors:** Jonathan Sherman, Maxim Y. Gorbunov, Oscar Schofield, Paul G. Falkowski

**Affiliations:** ^1^ Environmental Biophysics and Molecular Ecology Program, Department of Marine and Coastal Sciences, Rutgers The State University of New Jersey New Brunswick New Jersey USA; ^2^ Center for Ocean Observing Leadership, Department of Marine and Coastal Sciences Rutgers, The State University of New Jersey New Brunswick New Jersey USA

## Abstract

The West Antarctic Peninsula (WAP) is a highly productive polar ecosystem where phytoplankton dynamics are regulated by intense bottom‐up control from light and iron availability. Rapid climate change along the WAP is driving shifts in the mixed layer depth and iron availability. Elucidating the relative role of each of these controls and their interactions is crucial for understanding of how primary productivity will change in coming decades. Using a combination of ultra‐high‐resolution variable chlorophyll fluorescence together with fluorescence lifetime analyses on the 2017 Palmer Long Term Ecological Research cruise, we mapped the temporal and spatial variability in phytoplankton photophysiology across the WAP. Highest photosynthetic energy conversion efficiencies and lowest fluorescence quantum yields were observed in iron replete coastal regions. Photosynthetic energy conversion efficiencies decreased by ~ 60% with a proportional increase in quantum yields of thermal dissipation and fluorescence on the outer continental shelf and slope. The combined analysis of variable fluorescence and lifetimes revealed that, in addition to the decrease in the fraction of inactive reaction centers, up to 20% of light harvesting chlorophyll‐protein antenna complexes were energetically uncoupled from photosystem II reaction centers in iron‐limited phytoplankton. These biophysical signatures strongly suggest severe iron limitation of photosynthesis in the surface waters along the continental slope of the WAP.

Iron availability limits phytoplankton growth and production across ~ 30% of the ocean's surface (Moore et al. 2013). However, iron requirements vary dramatically among species (Ho et al. 2003) and phytoplankton communities may remain relatively iron replete even in regions with extremely low concentrations of iron, such as the South Pacific Gyre (Bonnet et al. 2008). Consequently, there is a need to develop sensitive diagnostic tools for iron limitation in phytoplankton (Hopkinson et al. 2007; Behrenfeld and Milligan 2013). The Southern Ocean has garnered particular interest as it is the world's largest iron‐limited region (Boyd 2002; Strzepek et al. 2012).

Over several decades, variable fluorescence signals from photosystem II (PSII) have been used to measure photosynthetic conversion efficiencies. This efficiency, commonly denoted as F_v_/F_m_, is the quantum yield of photochemistry in PSII (Φ_PSII_); that is, the ability of absorbed light to drive photosynthetic electron transport from water to a terminal electron acceptor (Kolber et al. 1998). The rate of induction of variable fluorescence on the microsecond time scale can also be used to calculate the effective absorption cross section of PSII (*σ*_PSII_) (Kolber et al. 1998; Gorbunov and Falkowski 2005). This latter parameter is a product of the optical absorption cross section of PSII (i.e., the size of the PSII antennae) and the quantum yield of photochemistry in the reaction center (RC) (Ley and Mauzerall 1982; Kolber et al. 1998; Falkowski et al. 2004; Falkowski and Raven 2014).

Fluorescence emission and nonradiative thermal dissipation (with the quantum yields of Φ_F_ and Φ_T_, respectively) compete with photochemistry to dissipate absorbed photons (Butler and Strasser 1977; Butler 1978; Falkowski et al. 2017). The three are complementary, meaning the sum of the three yields is 1.00 (Butler 1978). Moreover, they are remarkably sensitive to the effects of iron limitation on phytoplankton physiology (Lin et al. 2016). Extensive measurements of variable fluorescence under iron limitation have revealed substantial decreases in maximal Φ_PSII_ and pronounced increases in *σ*_PSII_. These responses were observed in cultures (Greene et al. 1991; Vassiliev et al. 1995; Strzepek et al. 2012, 2019) and in situ (Greene et al. 1994; Gervais et al. 2002; Suzuki et al. 2002; Behrenfeld and Milligan 2013). Furthermore, shipboard and in situ iron enrichment experiments, revealed rapid increases in F_v_/F_m_ and decreases in *σ*_PSII_ following iron amendment (Gervais et al. 2002; Hutchins et al. 2002; Coale et al. 2004; Hopkinson et al. 2007; Moore et al. 2007; Ryan‐Keogh et al. 2017).

Low Φ_PSII_ reflects a downregulation in functional RCs, complemented by an increased pool of light harvesting complexes (LHC), some energetically uncoupled from the RCs (Greene et al. 1991; Schrader et al. 2011; Macey et al. 2014). The LHCs that are still coupled energetically serve fewer functional RCs, resulting in an increased *σ*_PSII_. With this, phytoplankton economize the high iron quota of RCs (Strzepek et al. 2012). At low light, large LHCs increase excitation energy loss through thermal dissipation and fluorescent emission before being trapped in an active RC (Wientjes et al. 2013). In the Southern Ocean, Strzepek et al. (2019) proposed the low temperatures mitigate this loss. Conversely, at saturating light, the few active RCs are subjected to overexcitation and damage (Greene et al. 1992). To cope, iron‐limited phytoplankton increase rapid nonphotochemical quenching (NPQ) components (Petrou et al. 2011; Alderkamp et al. 2013). NPQ represents a suite of photoprotective mechanisms activated at high light, effectively increasing Φ_T_ and simultaneously decreasing *σ*_PSII_ (Goss and Lepetit 2015; Kuzminov and Gorbunov 2016; Buck et al. 2019). Further work is needed to rapidly assess the occurrence and function of these physiological responses to iron limitation in natural assemblages (Behrenfeld and Milligan 2013). However, for a truly comprehensive evaluation, an additional yield needs to be measured alongside Φ_PSII_. Previous studies have suggested methods to derive additional yields from variable fluorescence (Hendrickson et al. 2004; Kramer et al. 2004). However, these methods critically depend on a priori assumptions regarding the antenna‐RC organization.

To that end, we developed an extremely sensitive, sea‐going instrument, PicoLiF (Picosecond Lifetime Fluorescence), which continuously measures in situ chlorophyll fluorescence lifetimes in the picosecond time domain. When the measured lifetimes are normalized to the natural lifetime (15,000 ps, or 15 ns in the case of chlorophyll *a* [Chl *a*]; Brody and Rabinowitch 1957), the result is the quantum yield of fluorescence, Φ_F_. As all three quantum yields sum to unity, direct measurements of Φ_PSII_ and Φ_F_ allow quantification of Φ_T_ by difference (Lin et al. 2016) and the fraction of energetically uncoupled LHC‐RC complexes (Park et al. 2017). In addition, they provide insight into regulation of energy transfer (Buck et al. 2019) and photoprotection (Kuzminov and Gorbunov 2016) in PSII. In the oceans, Φ_F_ varies about fivefold in response to light and nutrients (Lin et al. 2016). Indeed, the direct measurement of Φ_F_ from lifetimes in the picosecond time domain is the only way to calibrate or verify remotely sensed Φ_F_, which is a highly derived product (Huot et al. 2005; Behrenfeld et al. 2009; Lin et al. 2016).

Here, we evaluated surface phytoplankton photophysiology in the West Antarctic Peninsula (WAP). In this region, a cross shelf iron gradient exists, hypothesized to control phytoplankton abundance and productivity (Annett et al. 2017). Custom‐built fluorometers were deployed during the 2017 annual WAP Long Term Ecological Research cruise in the austral summer. A FIRe (Fluorescence Induction and Relaxation) instrument measured F_v_/F_m_, *σ*_PSII_. Simultaneously, the PicoLiF instrument measured fluorescence lifetimes. We hypothesized our combined measurements would reveal a distinct iron‐limited physiology, with significantly higher Φ_T_, and an increased pool of uncoupled LHC‐RC in the WAP offshore waters.

## *Materials and methods*

### Study area

**Fig 1:**
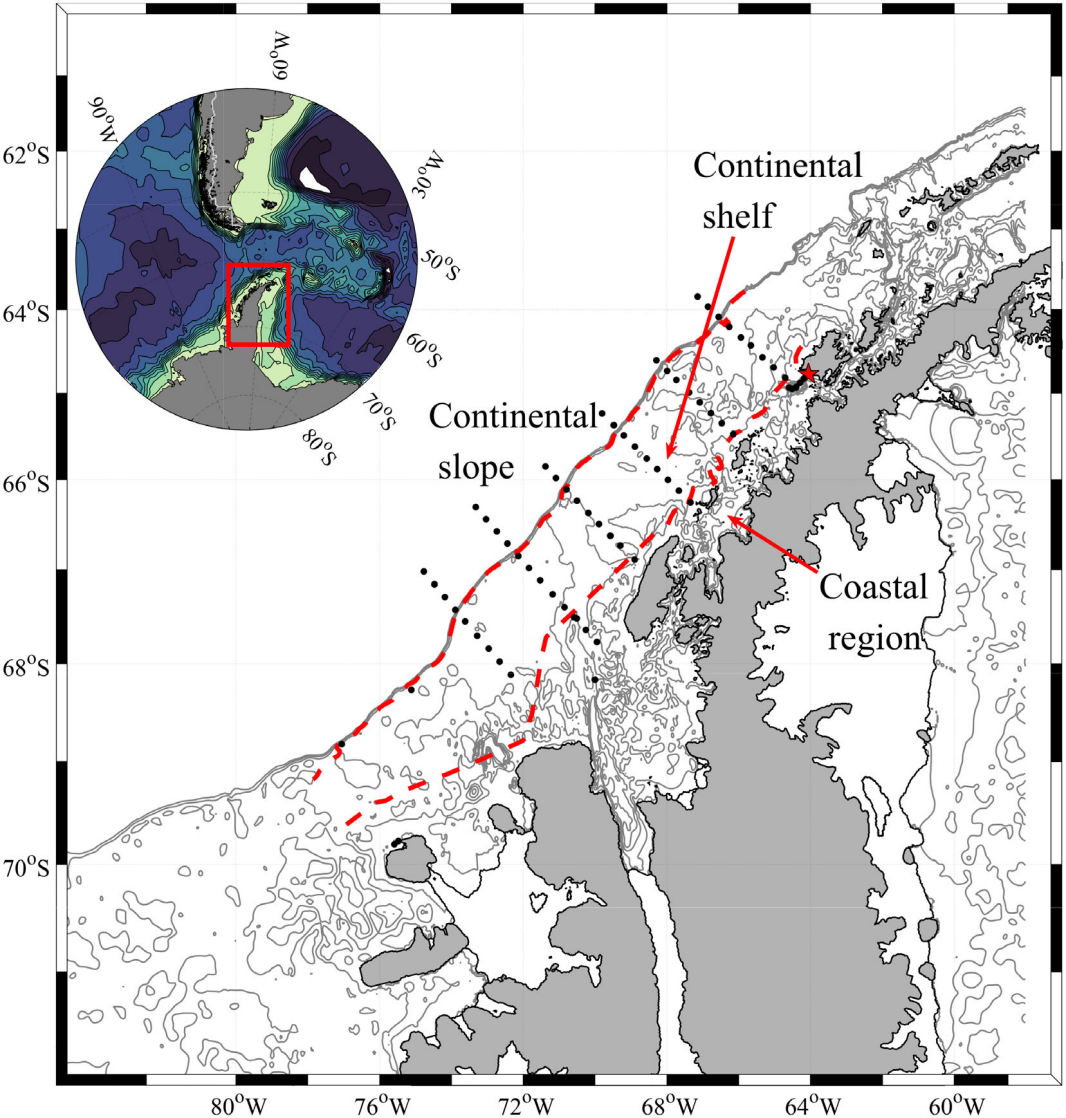
West Antarctic Peninsula (WAP) Long Term Ecological Research (LTER) site. Insert at top left shows the WAP in relation to South America. Black dots on main figure represent the sampling stations along the 100 line in the south to the 600 line in the north. Red dashed lines denote the three subregions along the WAP; the coast, the continental shelf and the continental slope. Red star is the location of the U.S Palmer station on Anverse Island. Bathymetry in figure and insert is from ETOPO1 dataset.

Data were collected on board the ASRV Laurence M. Gould. Sampling was carried out along perpendicular cross shelf transects spaced 100 km apart. The study region corresponds to the LTER project grid lines 100–600 (Waters and Smith 1992) (Fig. 1). Following Steinberg et al. (2015), we differentiate between three subregions across the WAP: the shallow coastal region, the continental shelf, and the deep continental slope roughly 200 km offshore.

### Sample collection and analysis

Sampling stations were at 20 km intervals along each grid line. Samples were collected for Chl *a* and dissolved inorganic nutrients (nitrate, phosphate, and silicate) following Carvalho et al. (2019). Variable fluorescence and fluorescence lifetime data were collected continuously from surface waters (~ 5 m) while underway with FIRe and PicoLiF fluorometers respectively, as described by Lin et al. (2016). The instruments used flow through cuvettes connected to the ship's surface water intake pump. The water passed through two de‐bubblers prior to entering the cuvette.

Mixed layer depth (MLD) was defined as the depth at which the maximum buoyancy frequency was observed in CTD profiles, following Carvalho et al. (2017). The critical depth was calculated from surface daily integrated photosynthetically available radiation (PAR), collected from the mast of the ship (QSR‐240P, Biospherical Instruments) and the light attenuation coefficient (K_PAR_) calculated from the empirical relationship of K_PAR_ and Chl *a* concentration, as proposed by Sverdrup (1953) and Nelson and Smith (1991). Night and day were differentiated using NOAA's solar calculator (https://www.esrl.noaa.gov/gmd/grad/solcalc/).

### Photophysiology

We recorded variable fluorescence using a mini‐FIRe instrument as previously described (Gorbunov and Falkowski 2005; Kuzminov and Gorbunov 2016). Variable fluorescence was induced by a saturating single turnover flash (STF) from blue light‐emitting diodes (450 nm with 30 nm half bandwidth), which cumulatively reduce all PSII RCs within ca. 100 *μ*s. This excitation protocol results in minimum and maximum fluorescence yields (F_0_ and F_m_). The quantum yield of photochemistry in PSII was then calculated as (F_m_−F_0_)/F_m_ = F_v_/F_m_ (Butler 1978; Kolber et al. 1998). The effective absorption cross section of PSII, *σ*_PSII_ (at 450 nm), is calculated by fitting the fluorescence rise to a cumulative one‐hit Poisson function (Ley and Mauzerall 1982).

Every ~ 30 min the flow through on the mini‐FIRe was automatically paused in order to conduct slow fluorescence irradiance (FE) curves. These were used to retrieve electron transport rates (ETR) as a function of irradiance and to characterize the state of phytoplankton photoacclimation to their short‐ and long‐term light history (Falkowski 1994; Ralph and Gademann 2005). During FE curves, the water sample was trapped in the cuvette for ca. 10 min and then exposed to increasing PAR levels (0–800 *μ*mol photons m^−2^ s^−1^) with an actinic blue light source (450 nm). Every light step lasted 30–40 s to promote short‐term acclimation to each new PAR level, followed with standard measurements. FE curves in this study are termed slow as light steps were longer than other comparable studies, where light steps lasted 10–20 s (Serôdio et al. 2006; Suggett et al. 2015). This was done, as the acclimation is slower at lower water temperatures. From FE curves, we calculated the rate of photosynthetic electron transport normalized per PSII RC (ETR_PSII_, with units of e^−^ s^−1^ RC^−1^), as a function of PAR (Gorbunov et al. 2000, 2001) from

(1) $${\mathrm{ETR}}_{\text{PSII}}=E\times \sigma_{\text{PSII}}\times \left[\left(\Delta F^{\prime }/F_{m}^{\prime }\right)/\left(F_{v}/F_{m}\right)\right]$$

Here, E is irradiance, F_v_/F_m_ and *σ*_PSII_ are measurements in the dark (PAR = 0), respectively. $\Delta F^{\prime }/F_{m}^{\prime }$ is the quantum yield of photochemistry at a given PAR level, with the prime notation indicating measurement under ambient light ($\Delta F^{\prime }=F_{m}^{\prime }-F\prime$, where F′ is a steady‐state fluorescence at a given light step). FE curves were than fitted to a hyperbolic tangent function to derive maximal ETR through PSII (${\mathrm{ETR}}_{\text{PSII}}^{\max}$), and the E_K_ value (saturating light level) following Jassby and Platt (1976) as

(2) $${\mathrm{ETR}}_{\text{PSII}}={\mathrm{ETR}}_{\text{PSII}}^{\max}\times \tanh\left(E/E_{K}\right)$$

Picosecond fluorescence decays, measured with the PicoLiF, were deconvoluted from the instrument response function and fitted to a sum of three exponentials with a custom TCSPFIT Matlab package utilizing a Nelder‐Meade simplex algorithm (Enderlein and Erdmann 1997). Φ_F_ was then calculated from

(3) $$\Phi_{F}=\tau /\tau_{0}$$

where *τ* is the measured lifetime and *τ*_0_ is the natural lifetime of Chl *a* (Brody and Rabinowitch 1957; Brody 2002). The natural lifetime is the time that would be required for a molecule to return to the ground state from an excited state if fluorescence were the sole dissipation pathway. For Chl *a*, *τ*_0_ is 15 ns and is constant, independent of solvent, organism or environmental condition (Brody and Rabinowitch 1957; Brody 2002; Lakowicz 2006). We then calculated the quantum yield for thermal dissipation (Φ_T_) as

(4) $$\Phi_{T}=1-\left[\frac{F_{v}}{F_{m}}+\frac{\tau }{\tau_{0}}\right]$$

All fluorescence measurements were corrected for the blank signal measured routinely from filtered seawater (0.2 *μ*m) (Bibby et al. 2008). To minimize changes in temperature, the flow through system relies on thick walled tubing for insolation.

In the current setup, phytoplankton experienced ~ 10 min of low‐light acclimation from the time they entered the ship's underway system to when the FIRe and PicoLiF measurements were conducted. In this time frame, most of the rapid NPQ mechanisms (e.g., xanthophyll cycling) relax, leading to a recovery in F_v_/F_m_. However, this time is not sufficient to alleviate the effects of photoinhibition, which requires > 10 min to recover (Alderkamp et al. 2013). Considering this, our standard measurements represent a state in which phytoplankton are not in an entirely low‐light acclimated state. In effect, this means that F_v_/F_m_ may be slightly underestimated when the measurements are conducted during the day. This issue is more pertinent for the calculation of ETR, as the underlying assumption relies on a truly low‐light acclimated baseline (Eq. 1). As there is an additional low‐light acclimation period before a FE curve is measured the deviations mentioned above would decrease. In any case, from Eq. 1 it can be seen that underestimating F_v_/F_m_ and *σ*_PSII_ results in an underestimation of ETR. To minimize any under‐ or overestimations, preferential weight is given to data collected during the night. Although data collected during the day may cause some issues in interpretation, we argue that the low‐light acclimation periods used here are sufficient nonetheless to provide distinct differences in the photophysiological status of phytoplankton in response to iron and light availability in this region.

### Statistical analyses

The average of each photophysiological variable reported in this paper represents the median value. The median was chosen because each individual variable was not normally distributed and included statistical outliers. To describe the deviation from the median, we calculated the median absolute deviation, a robust measure of dispersion around the median (Leys et al. 2013). For improved spatial comparison of variables, surface maps were produced using a Locally Weighted Scatter‐plot Smoother. Significance with a *ρ* ≤ 0.05 was determined from Pearson's linear correlation.

## *Results*

### WAP physical and chemical setting

**Fig 2:**
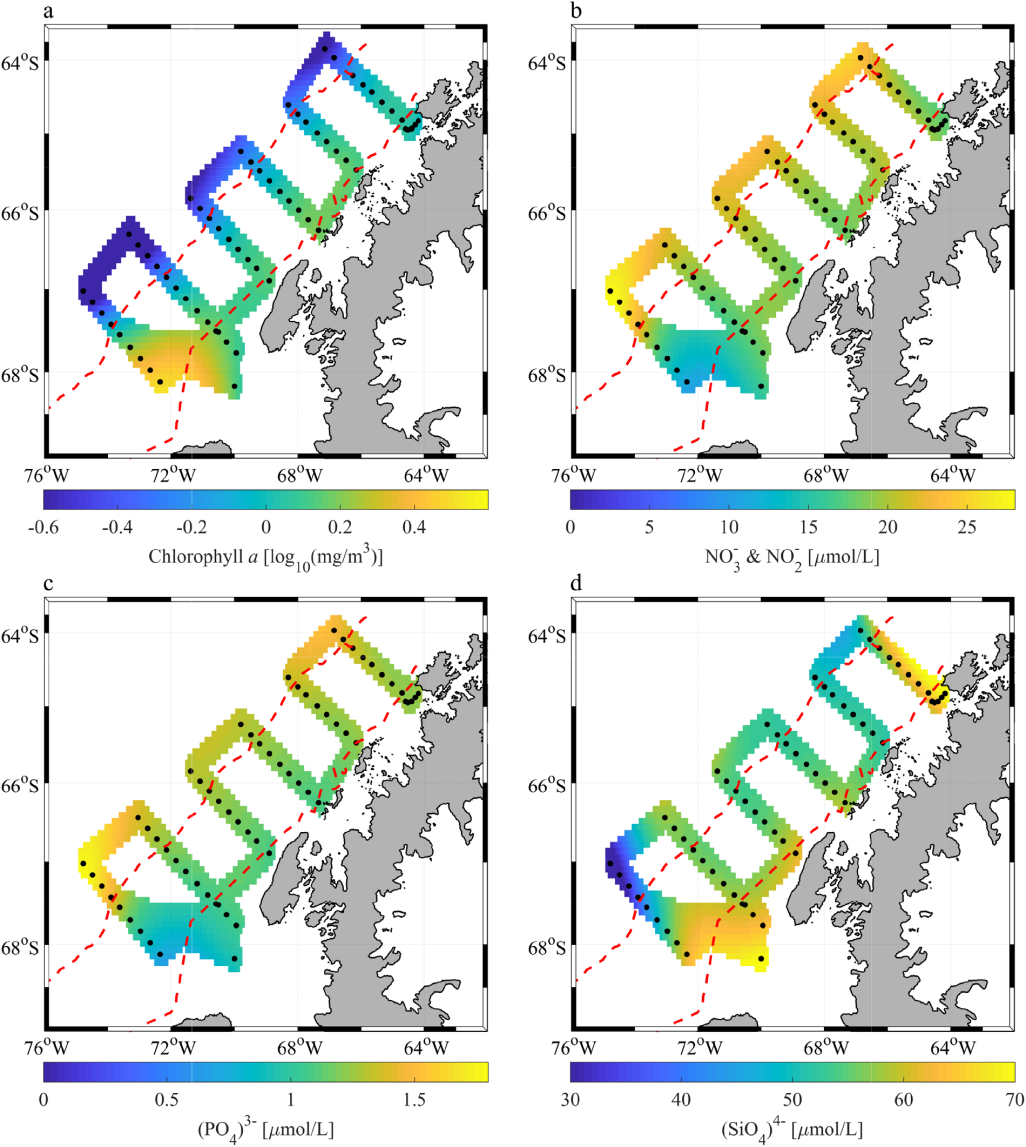
Surface distribution maps of; (**a**) Chl *a*, (**b**) nitrate and nitrite c‐phosphate, and (**d**) silicate. Data collected from CTD rosette cast or underway water collection, denoted by black dots. Red dashed lines highlight the borders between the WAP coast, shelf and slope regions. Note that Chl *a* is presented on a logarithmic scale to accommodate a range in orders of magnitude.

Surface concentrations of Chl *a* ranged from 0.06 to 16.9 mg m^−3^. Surface Chl *a* concentration significantly correlated with distance to shore (Table 1), with higher concentrations along the coast, decreasing offshore over the continental shelf and slope (Fig. 2a). Macronutrients were replete along the entire grid, with increasing nitrogen (as nitrate) and phosphate over the slope (Fig. 2b,c). Furthermore, N/P ratio tracked closely with the canonical 16/1 Redfield ratio, indicating that nitrogen and phosphate were not limiting factors in this region. Silicate, a crucial nutrient for diatoms, also decreased but was still abundant nonetheless (Fig. 2d). Overall, our data support the notion that along the WAP, oceanic conditions transition over a short distance (~ 200 km), from a coastal‐ to an HNLC‐ecosystem over the continental slope, where previous studies indicate iron depletion (Annett et al. 2017).

**Table 1:** Pearson's linear correlation coefficients matrix between independent variables (surface PAR and distance to shore) and dependent variables (F_v_/F_m_, fluorescence lifetime, Φ_T_, *σ*_PSII_, surface Chl *a* concentration, and ${\mathrm{ETR}}_{\max}^{\text{PSII}}$). All coefficients are significant (*ρ* ≪ 0.05).

	Surface PAR	Distance to shore
F_v_/F_m_	−0.444	−0.614
Fluorescence lifetime	−0.481	0.644
Φ_T_	0.435	0.653
σ_PSII_	−0.237	0.624
Surface Chl *a* conc.	0.089	−0.406
${\mathrm{ETR}}_{\text{PSII}}^{\max}$	0.522	0.319

### Spatial variability in photophysiological parameters

**Fig 3:**
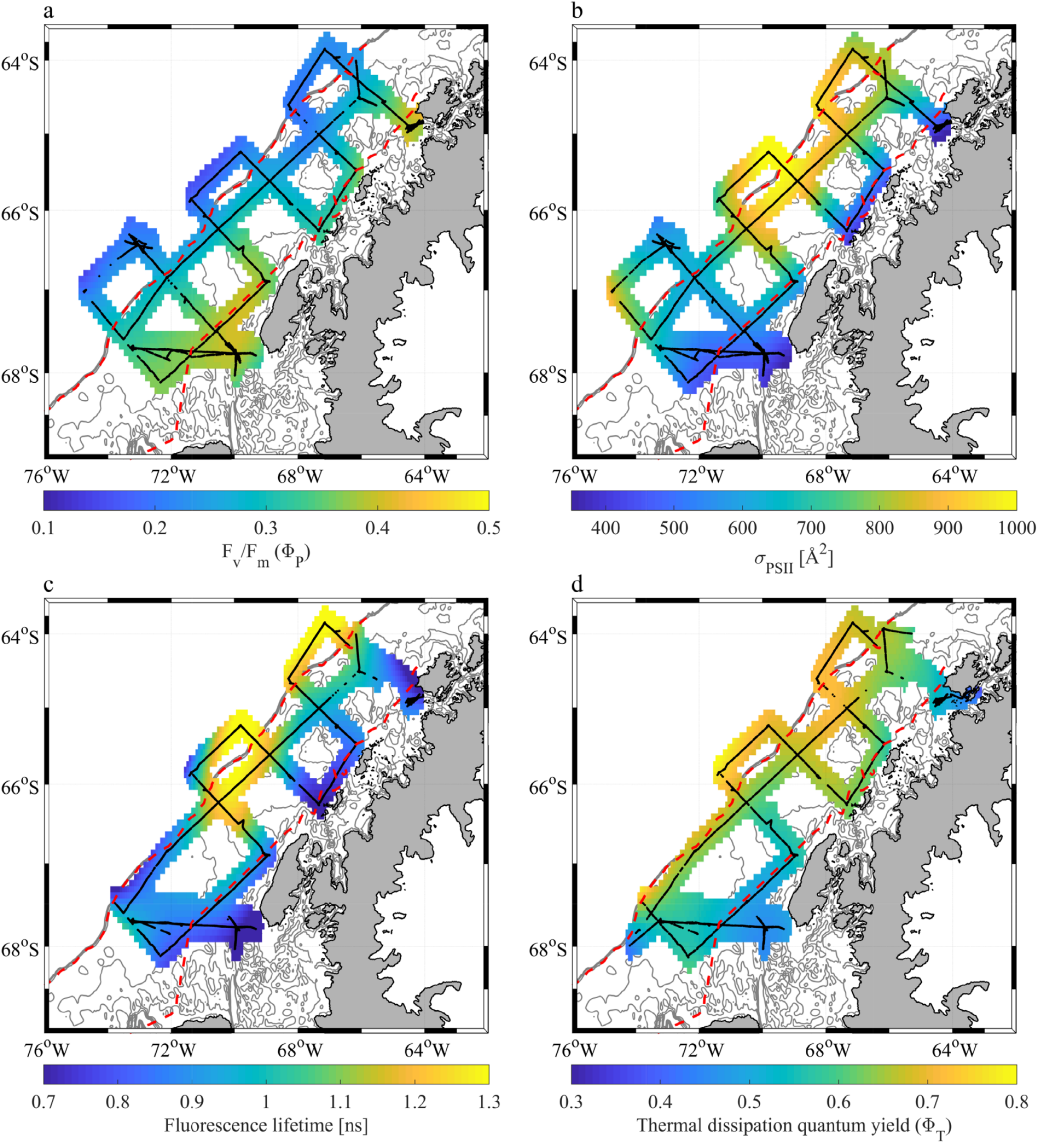
Surface distribution maps of; (**a**) F_v_/F_m_, (**b**) σ_PSII_, (**c**) fluorescence lifetime, and (**d**) thermal dissipation quantum yield (Φ_T_). F_v_/F_m_ and σ_PSII_ collected from underway FIRe measurements. Fluorescence lifetime collected from underway PicoLiF measurements. Φ_T_ calculated from Eq. 4. Black dots denote measurement locations. Red dashed lines highlight the borders between the WAP coast, shelf and slope regions.

Surface maps of F_v_/F_m_, fluorescence lifetimes, *σ*_PSII_ and Φ_T_ show clear gradients across the continental shelf (Fig. 3; Table 2). The differences are significant, showing the highest degree of correlation with distance from shore (Table 1). As seen in Table 2, throughout a diel cycle, F_v_/F_m_ values along the coast were relatively high (0.42 ± 0.06), and progressively decreased by up to 50% offshore along the shelf (0.3 ± 0.08) and slope (0.22 ± 0.04). Fluorescence lifetimes, in contrast, increased offshore. Along the coast fluorescence, lifetimes were relatively low, 0.77 ± 0.07 ns, increasing to 1.03 ± 0.17 ns along the shelf and 1.29 ± 0.23 ns along the slope. Consequently, Φ_T_ increased from 0.5 ± 0.05 along the coast, to 0.62 ± 0.06 and 0.69 ± 0.04 at the shelf and slope. Similarly, *σ*_PSII_ increased from 484 ± 54 Å^2^ along the coast to 694 ± 132 Å^2^ over the shelf, reaching 760 ± 168 Å^2^ at the slope. Likewise, diel averaged ${\mathrm{ETR}}_{\max}^{\text{PSII}}$ rates progressively increased from 58 ± 107 e^−^ s^−1^ RC^−1^ along the coast to 137 ± 55 and 230 ± 105 e^−^ s^−1^ RC^−1^ out over the shelf and slope (Table 2).

**Table 2:** Median and median absolute deviation of photophysiological parameters; F_v_/F_m_, σ_PSII_, fluorescence lifetime, Φ_T_, and ${\mathrm{ETR}}_{\max}^{\text{PSII}}$. Data were parsed by location; coast, shelf and slope, and by time; night or day.

		Night	Day	Full diel cycle
F_v_/F_m_	Coast	0.48 ± 0.03	0.39 ± 0.07	0.42 ± 0.06
	Shelf	0.35 ± 0.07	0.27 ± 0.08	0.3 ± 0.08
	Slope	0.24 ± 0.03	0.19 ± 0.04	0.22 ± 0.04
	Full grid	0.38 ± 0.1	0.29 ± 0.1	0.31 ± 0.1
σ_PSII_ [Å^2^]	Coast	476 ± 42	492 ± 58	484 ± 54
	Shelf	710 ± 142	678 ± 125	694 ± 132
	Slope	792 ± 174	744 ± 162	760 ± 168
	Full grid	588 ± 126	564 ± 120	568 ± 124
Fluorescence lifetime (ns)	Coast	0.81 ± 0.08	0.76 ± 0.1	0.77 ± 0.09
	Shelf	1.11 ± 0.13	0.97 ± 0.18	1.03 ± 0.17
	Slope	1.5 ± 0.06	1.07 ± 0.21	1.29 ± 0.23
	Full grid	1.03 ± 0.21	0.84 ± 0.15	0.9 ± 0.17
Φ_T_	Coast	0.46 ± 0.02	0.53 ± 0.05	0.5 ± 0.05
	Shelf	0.58 ± 0.06	0.65 ± 0.07	0.62 ± 0.06
	Slope	0.66 ± 0.02	0.72 ± 0.09	0.69 ± 0.04
	Full grid	0.53 ± 0.08	0.61 ± 0.09	0.59 ± 0.09
${\mathrm{ETR}}_{\text{PSII}}^{\max}$ (e^−^ s^−1^ RC^−1^)	Coast	58 ± 15	119 ± 37	107 ± 39
	Shelf	59 ± 17	176 ± 53	137 ± 55
	Slope	208 ± 118	263 ± 100	230 ± 105
	Full grid	64 ± 21	149 ± 53	128 ± 55

### Diel variability in photophysiological parameters

**Fig 4:**
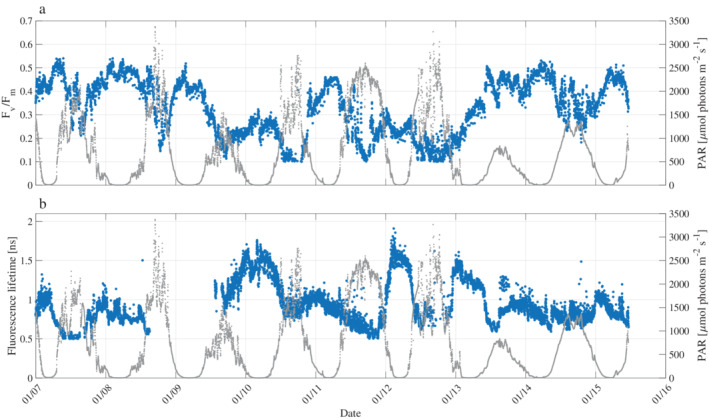
Diel cycles in (**a**) F_v_/F_m_ (blue) and (**b**) fluorescence lifetime [ns] (blue) on the left Y axis from underway FIRe and PicoLiF measurements. Right Y‐axis shows atmospheric PAR intensity (gray dots) as measured from the ships mast. Data represent the first week of the cruise.

**Fig 5:**
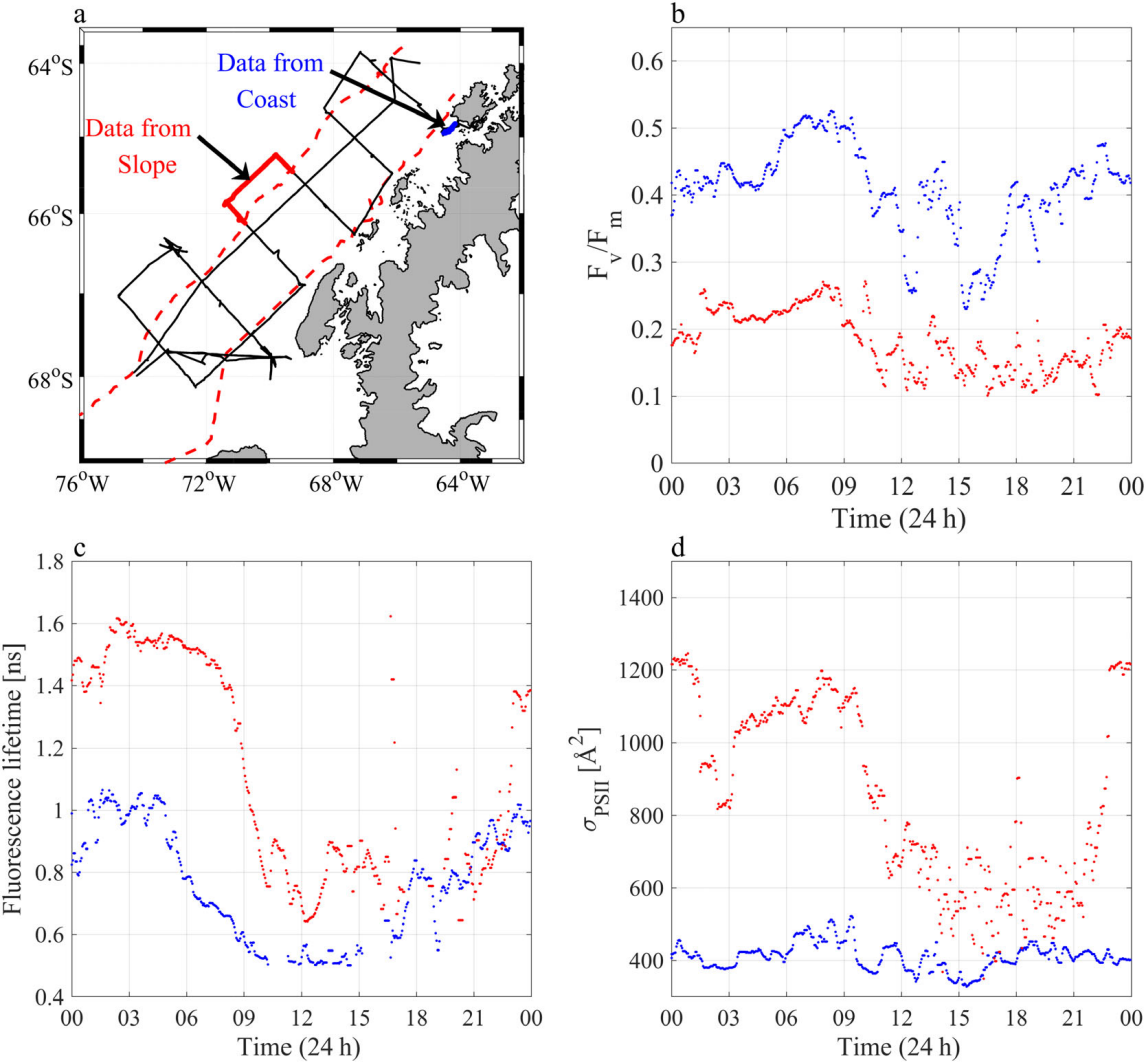
A comparison of diel cycles in phytoplankton photophysiology over the coast, in blue, and continental slope, in red. (**a**) Locations in which the data plotted were collected. (**b**) F_v_/F_m_, (**c**) fluorescence lifetime, and (**d**) *σ*_PSII_.

Both F_v_/F_m_ and fluorescence lifetimes displayed a pronounced diel cycle throughout the WAP (Fig. 4), negatively correlated with surface PAR (Table 1). Median F_v_/F_m_ values during the night across the whole grid were 0.38 ± 0.1, concurrent with an average fluorescence lifetime of 1.03 ± 0.21 ns. During the day, as light intensity increased, F_v_/F_m_ decreased by ~ 20% to 0.29 ± 0.1, while fluorescence lifetimes decreased to 0.84 ± 0.15 ns. Conversely, Φ_T_ increased from 0.53 ± 0.08 to 0.61 ± 0.09 between night and day (Table 2). A weak negative correlation between *σ*_PSII_ and PAR intensities was seen (Table 1). However, despite this, a diel cycle was not a prominent feature, particularly along the coast (Fig. 5d; Table 2), and *σ*_PSII_ values during the night and day averaged 588 ± 126 Å^2^ and 564 ± 120 Å^2^, respectively (Table 2). On the other hand, ${\mathrm{ETR}}_{\max}^{\text{PSII}}$ correlated positively with PAR (Table 1). During the night ${\mathrm{ETR}}_{\max}^{\text{PSII}}$ was low (64 ± 21 e^−^ s^−1^ RC^−1^), while during the day ${\mathrm{ETR}}_{\max}^{\text{PSII}}$ more than doubled (149 ± 53 e^−^ s^−1^ RC^−1^) (Table 2).

## *Discussion*

**Fig 6:**
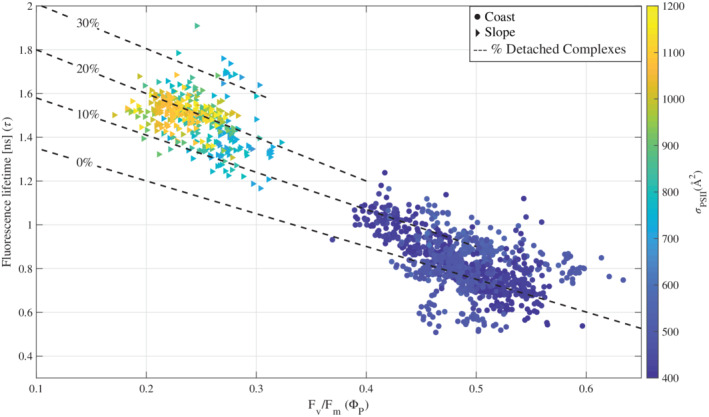
Relationship between F_v_/F_m_, fluorescence lifetime and *σ*_PSII_ (in color bar). Circles represent data collected from the coast and triangles data from the slope. In both locations, only data collected during the night are shown in order to remove the nonlinear effect of NPQ on the otherwise linear relationship between F_v_/F_m_ and fluorescence lifetime (Lin et al. 2016). Dashed lines indicate the fraction of uncoupled reaction centers. 0% dashed lines represents the modeled linear dependency of F_v_/F_m_ and fluorescence lifetime (Butler 1978). 10–30% dashed lines represent the dependency of these parameters in detached antenna PSII reaction centers complexes.

**Fig 7:**
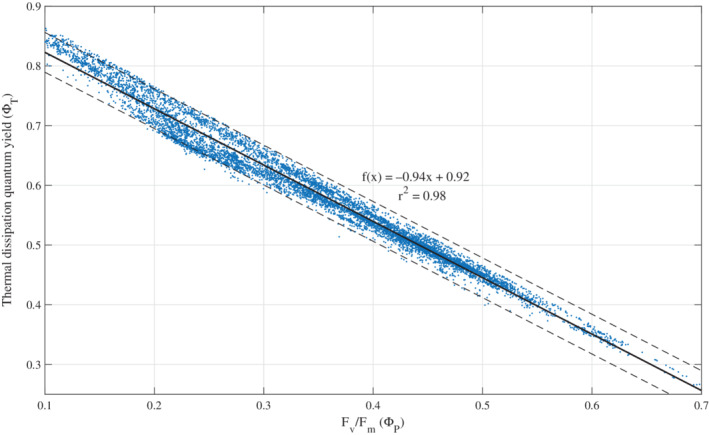
Relationship between F_v_/F_m_ and the quantum yield of thermal dissipation (Φ_T_). The quantum yield of thermal dissipation is calculated from underway measurements of variable fluorescence with a FIRe instrument and fluorescence lifetime with a PicoLiF instrument as $\Phi_{T}=1-\left[\left(\frac{\mathrm{Fv}}{\mathrm{Fm}}\right)+\left(\frac{\tau }{\tau_{0}}\right)\right]$, where *τ* is the measured lifetime in ns and *τ*_0_ is the natural lifetime of Chl *a*, 15 ns (*see* main text). A linear regression line, the 95% confidence interval, the regression equation, and the *r* ^2^ are indicated (*n* = 7590).

Our results reveal a clear gradient in photophysiological characteristics across the continental margin of the WAP (Fig. 3; Table 2). This gradient is consistent with bottom‐up control by iron availability in surface waters and supports the hypothesis that iron strongly limits phytoplankton photochemical energy conversion offshore (Annett et al. 2017; Schofield et al. 2018). The combination of variable fluorescence and lifetimes revealed an increased amount of uncoupled LHC complexes under iron limitation (Fig. 6) as well as a clear tradeoff between photochemistry and thermal dissipation (Fig. 7), resulting from the spatial gradient in iron stress across the WAP. To support this conclusion, we discuss a number of physiological responses to iron limitation across the WAP. These include spatial variabilities in nighttime values and diel cycles of photophysiological parameters, LHC‐RC uncoupling, and Φ_T_. In addition, we examine the variability in ${\mathrm{ETR}}_{\max}^{\text{PSII}}$.

At night, when NPQ is nil (Lin et al. 2016), F_v_/F_m_ over the continental slope decreased by 50% in comparison to the iron richer regions closer to the coast (Table 2). At the same time, fluorescence lifetimes and *σ*_PSII_ increased by 85% and 65%, respectively. These trends are diagnostic of iron stressed photosynthesis along the continental slope. The extremely high values of *σ*_PSII_ offshore corroborate previous laboratory measurements on iron‐limited Southern Ocean species (Strzepek et al. 2019). Diel cycles of photophysiological parameters were similar across the WAP, with a nighttime maxima and midday minimum (Fig. 5). However, the magnitude of diel variations was much larger in iron‐limited regions (Fig. 5; Table 2). The diel cycles observed in the iron‐limited WAP continental slope contrast with previously established signatures of iron limitation observed in the Equatorial Pacific (Behrenfeld and Kolber 1999). In that region, dominated by cyanobacteria, F_v_/F_m_ decreased by 35–60% following the sunset and recovered at sunrise, resulting in a pillared nighttime feature. Behrenfeld and Kolber (1999) concluded this diel fluorescent pattern was due to state transitions in iron‐limited plankton. Such diel patterns were not observed in iron‐limited regions in the WAP (Figs4, 5). This is because phytoplankton assemblages in the WAP are dominated by diatoms (Schofield et al. 2017), in which state transitions are absent (Owens 1986).

We next consider the occurrence of energetically uncoupled LHC complexes. The basic biophysical model for energy distribution in the photosynthetic unit predicts an inverse relationship between low‐light acclimated Φ_PSII_ and Φ_F_ (Butler 1978; Lin et al. 2016; Falkowski et al. 2017). The correlation between F_v_/F_m_ and fluorescence lifetime collected at night validates our assumption of linearity in these two yields (Pearson's linear correlation coefficient of −0.91, *ρ* < 0.05). However, the data are not consistent with Butler's model (Fig. 6). To calculate the fraction of uncoupled LHC complexes, the relationship between F_v_/F_m_ and fluorescence lifetime was modeled for three physiological states with different lifetimes (Park et al. 2017). Two states represent cases in which LHC complexes are coupled to RCs and RCs are fully open or fully closed with lifetimes of 0.5 and 1.5 ns, respectively. The third represents uncoupled LHCII complexes with a very long lifetime of 4 ns (Palacios et al. 2002). The presence of such energetically detached antenna complexes would ultimately lead to longer measured lifetimes, and these lifetimes may exceed the values observed for fully closed RCs (~ 1.5 ns).

In Fig. 6, nighttime F_v_/F_m_ values are plotted against lifetimes. A distinct deviation from the classical inverse relationship predicted by Butler's model is seen at the low F_v_/F_m_. Moreover, two clusters are clearly seen. The first cluster represents coastal data, with high F_v_/F_m_, low fluorescence lifetimes and small *σ*_PSII_. This coastal cluster aligns fairly well with the modeled case for open RC with nearly fully coupled antenna complexes, as expected for iron‐replete conditions. The second cluster represents data from the continental slope, with low F_v_/F_m_ and long fluorescence lifetimes. This analysis suggests that 20–30% of antenna complexes are detached in iron‐limited waters offshore.

Over the WAP slope, Φ_T_ significantly increased (Fig. 3d; Table 2), confirming our hypothesis that phytoplankton increase Φ_T_ as iron limitation intensifies. At night, in the absence of NPQ, Φ_T_ along the slope was ~ 45% higher than in coastal waters (Table 2). This increase in Φ_T_ is driven by a reduction in the photosynthetic use efficiency. With few active RC, and a significantly large and uncoupled LHC, excitons are more likely to dissipate as heat (Strzepek et al. 2019). In addition to the positive correlation with distance to shore, Φ_T_ also positively correlated with increasing light availability (Table 1). During the day, Φ_T_ increased by an additional 15% across the WAP (Table 2), yet offshore values were still ~ 40% higher than onshore values. This daytime increase in Φ_T_ indicates NPQ activation that effectively increases thermal dissipation. As iron‐stressed phytoplankton are more prone to photooxidative damage at the active RCs (Greene et al. 1992; Strzepek et al. 2012), increasing Φ_T_ acts to further decrease excitation pressure on the RC in favor of thermal dissipation in the uncoupled antenna complex. To examine this hypothesis, we plotted the relationship between F_v_/F_m_ and thermal dissipation from the full dataset (Fig 7). A liner regression revealed a slope of −0.94 (*r* ^2^ = 0.98); the deviation from a −1.00 slope is attributed to ca. 6% dissipation by fluorescence (Lin et al. 2016). The increased Φ_T_ observed in the WAP supports recent studies showing higher photoprotective capacities in iron‐limited phytoplankton assemblages (Alderkamp et al. 2013; Schallenberg et al. 2020).

Moreover, the increase in uncoupled complexes combined with exceptionally high Φ_T_ offshore strongly agrees with the proposed mechanism for efficient NPQ in diatoms. This mechanism attributes the rapid NPQ capacity to thermal dissipation in the LHC, driven by xanthophyll pigment cycling and the presence of Lhcx proteins (Lepetit et al. 2017; Buck et al. 2019). These presumably cause a conformational change that distances the antenna complex from the RC, increasing the Föster resonance energy transfer distance, functionally mediating the energetic uncoupling of the LHC and RC.

Lastly, we turn to evaluate possible light limitation in the WAP surface waters, a second, potentially important bottom‐up control in this region (Moline 1998). Increased photoprotective activity during the day across the WAP, inferred from Φ_T_ (Table 2), suggests that phytoplankton are exposed to saturating light intensities in the near‐surface layer. Combined with the high ${\mathrm{ETR}}_{\max}^{\text{PSII}}$ values measured during the day (Table 2), it is highly unlikely that surface phytoplankton were light limited.

Although ${\mathrm{ETR}}_{\max}^{\text{PSII}}$ was initially assessed in regard to light limitation, it was surprising and perhaps counterintuitive to observe significantly higher (~ 120%) ${\mathrm{ETR}}_{\max}^{\text{PSII}}$ in the iron‐limited waters offshore (Table 2). A similar response in ${\mathrm{ETR}}_{\max}^{\text{PSII}}$ to iron limitation has been reported in phytoplankton assemblages from the Northeast subarctic Pacific, where iron amendment experiments resulted in decreased ${\mathrm{ETR}}_{\max}^{\text{PSII}}$ (Schuback et al. 2015). Similar results were seen in laboratory experiments with the diatom *Thalassiosira oceanica*, the haptophyte *Chrysochromulina polylepis* (Schuback et al. 2015), and the cyanobacterium *Synechococcus sp*. (Blanco‐Ameijeiras et al. 2018). The iron‐limited increase in ${\mathrm{ETR}}_{\max}^{\text{PSII}}$ is assumed to be an additional effect of the iron economizing physiology. In this manner, more excitons are funneled from the large antenna to fewer functional RC, leading to increased ETR per active PSII RC, each associated with a larger *σ*_PSII_. While we argue that our data provide little evidence for light limitation in the surface waters we measured, and is supported by previous studies (Moline et al. 1996), the effect of the MLD on light availability in the water column cannot be overlooked. Indeed, along the coast, MLD (13.3 ± 5.1) nearly reached the critical depth (14.9 ± 8.4 m), while along the slope, the MLD exceeded by up to ~ 30% the critical depth (30 ± 6 and 22.9 ± 10.9 m, respectively). Sverdrup's critical depth hypothesis (Sverdrup 1953) appears to imply that light limitation is particularly severe in the water column along the continental slope. Why then, is there little photophysiological evidence for light limitation in the surface waters offshore? We speculate that our data agree with the hypothesis that in Southern Ocean phytoplankton, the photophysiological response to iron limitation eliminates the antagonistic co‐limitation of iron and light (Strzepek et al. 2012). Accordingly, the high capacity for light harvesting in the iron‐limited slope community alleviates light limitation. On the other hand, in the iron replete coastal community, light limitation is more probable and agrees with a recent study along the coast (Carvalho et al. 2019). Still, during the day coastal phytoplankton in the surface waters themselves experience light saturating conditions. This may result from a long‐term acclimation to limiting light conditions in the water column, subjecting phytoplankton to overexcitation at saturating light, likely only met at the surface.

Our analysis assumes a uniform taxonomic composition across the WAP, which can potentially influence fluorescence measurements (Suggett et al. 2009). This is a fairly safe assumption as HNLC regions are anomalous in this respect, yet with relatively consistent fluorescence signatures across taxa (Suggett et al. 2009). This is further supported in Southern Ocean species (Strzepek et al. 2019), in particular diatoms, the dominant species in the WAP (Schofield et al. 2017).

Data presented here provide strong evidence for a distinct gradient in the degree of iron limitation across the WAP during the summer. Iron limitation was shown to be minimal at the coast and severe further offshore. As we hypothesized, combined measurements of Φ_PSII_ and Φ_F_ showed increased fractions of uncoupled LHC‐RC complexes as well as clear increases in Φ_T_ resulting from iron stress. The deep MLD across the WAP may have caused light limitation in the water column. Nonetheless, the clear acclimation to iron stress in the surface waters along the slope effectively reduced potential light limitation to a degree that phytoplankton were more susceptible to light saturation. Our in‐depth analysis of strictly biophysical mechanisms in response to iron stress is highly supported by a large number of studies, further strengthening our conclusions.

The WAPs case study, presented here, highlights the potential of our coupled Φ_PSII_ and Φ_F_ measurements as a rapid diagnostic tool for in situ assessments of iron limitation at high spatial and temporal resolution. More critically, this diagnostic tool provides a unique new avenue to assess in situ the role of uncoupled complexes in natural assemblages, their effect on satellite retrieved chlorophyll fluorescence and primary productivity models.

## Conflict of interest

None declared.

## Data Availability

Nutrient and Chlorophyll data are available through Palmer LTER Datazoo (https://oceaninformatics.ucsd.edu/datazoo/catalogs/pallter/datasets). Underway Fire and PicoLiF data deposited at https://seabass.gsfc.nasa.gov/.
